# 2291. SARS-CoV-2 Viral Kinetics Among Immunologically Naïve Adults: Comparison by Variant and Reinfection Status

**DOI:** 10.1093/ofid/ofad500.1913

**Published:** 2023-11-27

**Authors:** Margaret K Doll, Brianna Levenson Shakoor, Louise E Kimball, Jeffery Crukley, Nina Ozbek, Rachel L Blazevic, Larry Mose, Jim Boonyaratanakornkit, Terry L Stevens-Ayers, Kevin Cornell, Benjamin D Sheppard, Emma Hampson, Faria Sharmin, Benjamin Goodwin, Jennifer M Dan, Tom Archie, Terry O’Connor, Michael J Boeckh, Shane Crotty, Alpana Waghmare

**Affiliations:** Albany College of Pharmacy and Health Sciences, Albany, New York; La Jolla Institute for Immunology, La Jolla, California; Fred Hutchinson Cancer Center, Seattle, Washington; Data Science and Statistics, Toronto, Ontario, Canada; Fred Hutchinson Cancer Center, Seattle, Washington; Fred Hutchinson Cancer Center, Seattle, Washington; Fred Hutchinson Cancer Center, Seattle, Washington; Fred Hutchinson Cancer Center, Seattle, Washington; Fred Hutchinson Cancer Center, Seattle, Washington; St. Luke's Medical Center, Ketchum, Idaho; St. Luke's Medical Center, Ketchum, Idaho; St. Luke's Medical Center, Ketchum, Idaho; Albany College of Pharmacy and Health Sciences, Albany, New York; La Jolla Institute for Immunology, La Jolla, California; UCSD, La Jolla, California; St. Luke's Medical Center, Ketchum, Idaho; St. Luke's Wood River Medical Center, Ketchum, Idaho; Fred Hutchinson Cancer Center, Seattle, Washington; La Jolla Institute for Immunology, La Jolla, California; Fred Hutchinson Cancer Center/University of Washington, Seattle, Washington

## Abstract

**Background:**

SARS-CoV-2 viral dynamics offer insights into clinical trajectories and immune responses. While research has explored SARS-CoV-2 viral kinetics by variant and immune status, changes in population immunity make results difficult to interpret. Here, we examined SARS-CoV-2 viral kinetics in a community-based cohort of immunologically naïve adults.

**Methods:**

Unvaccinated adults 30 to 64 years of age without prior infection were followed for ≤ 72 weeks. Subjects submitted weekly nasal swabs for SARS-CoV-2 RT-PCR; if symptomatic or positive, swabs were collected every other day (up to 14 days). We examined RT-PCR cycle threshold (Ct) results from infections with sufficient data, defined as ≥ 3 swabs collected between ‒10 and 28 days of the peak Ct value with ≥ 1 Ct< 30. Bayesian hierarchical piecewise models were used to estimate viral kinetics by variant (classified by date) or first vs. second infections using data from peak swabs and those collected within ±3 days of another swab; if negative, Ct values were set to the detection limit and only the first of consecutive negatives were included.

**Results:**

Sufficient data were available for 179/187 (96%) first SARS-CoV-2 infections, with 27 (15%) Delta, 132 (74%) Omicron BA.1/BA.2, and 20 (11%) Omicron BA.4/BA.5 infections. Of these, 35 (20%) subjects had a second infection while unvaccinated (32 [91%] sufficient data). Figure 1 shows Ct values and model predictions by variant or infection status. Lower mean peak Ct values were found for first vs. second infections (‒5.7, 95% CI: ‒7.4, ‒4.1 cycles), and suggested for Delta vs. Omicron BA.1/BA.2 infections (‒1.4, 95% CI: ‒3.0, 0.5 cycles). Delta had a shorter mean time to peak (‒2.5, 95% CI: ‒3.8, ‒1.3 days) and longer clearance (2.7, 95% CI: 0.7, 4.9 days) vs. Omicron BA.1/BA.2 infections; first infections had longer clearance (3.5, 95% CI: 1.4, 5.3 days) vs. second infections.

**Figure 1.**
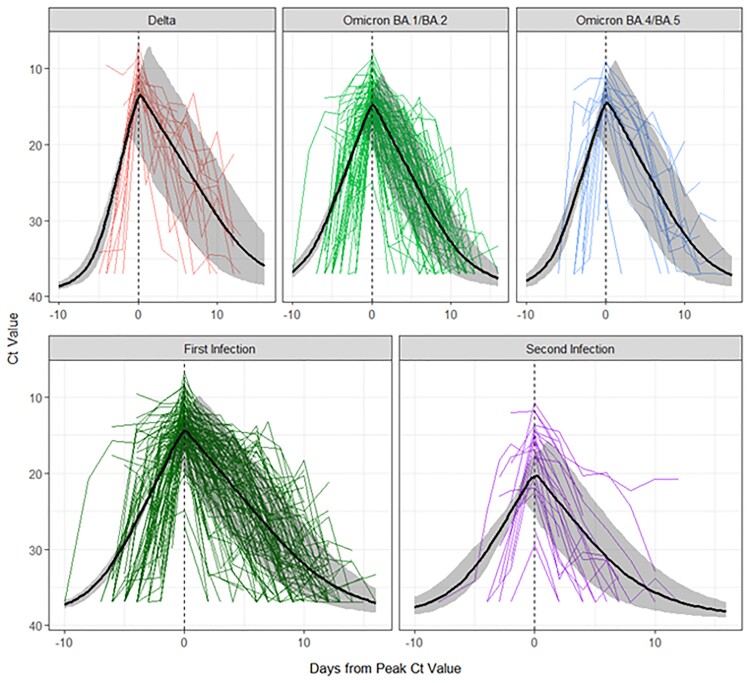
Participant RT-PCR cycle threshold (Ct) trajectories with model predictions (black lines) and 95% credible intervals (gray ribbons) by variant or infection status.

**Conclusion:**

Modeled estimates suggest Delta infections experienced higher viral loads, shorter time to peak, and longer clearance times compared with Omicron BA.1/BA.2. First infections had higher viral loads and longer clearance times vs. second infections. As population immunity is dynamic, characterizing viral kinetics among immunologically naïve individuals is valuable to inform SARS-CoV-2 trajectories.

**Disclosures:**

**Jim Boonyaratanakornkit, MD, PhD**, AstraZeneca: Advisor/Consultant|GlaxoSmithKline: Grant/Research Support|IgM Biosciences: Grant/Research Support|IgM Biosciences: Patent on antibodies to respiratory viruses|Vir Biotechnology: Grant/Research Support **Michael J. Boeckh, MD PhD**, Allovir: Advisor/Consultant|Amazon: Grant/Research Support|Ansun: Grant/Research Support|Merck: Advisor/Consultant|Merck: Grant/Research Support|Moderna: Advisor/Consultant|Symbio: Advisor/Consultant **Alpana Waghmare, MD**, Allovir: Grant/Research Support|Amazon: Grant/Research Support|Ansun Biopharma: Grant/Research Support|GlaxoSmithKline/Vir: Grant/Research Support|Kyorin Pharmaceuticals: Advisor/Consultant|Pfizer: Grant/Research Support|Vir Biotechnology: Advisor/Consultant

